# Serum sclerostin levels as a diagnostic marker for osteoporosis

**DOI:** 10.6026/973206300200054

**Published:** 2024-01-31

**Authors:** Modagan Paranthaman, K.S.V. Angu Bala Ganesh, Santhi Silambanan, Kuzhandai Velu Venkatapathy

**Affiliations:** 1 Department of Biochemistry, Dhanalakshmi Srinivasan Medical College and Hospital, Affiliated to The Tamilnadu Dr MGR Medical University, Perambalur 621 113, Tamil Nadu, India; 2 Department of Anatomy, Gujarat Adani Insitute of Medical Science, Bhuj, Gujarat 370001, India; 3 Department of Biochemistry, Sri Ramachandra Medical College, Sri Ramachandra Institute of Higher Education and Research, Porur, Chennai 600 116, Tamil Nadu, India; 4 Department of Biochemistry, Arunai Medical College and Hospital, Thenmathur, Tiruvannamalai 606603, India

**Keywords:** Osteoporosis, osteopenia, BMD, sclerostin

## Abstract

Osteoporosis is asymptomatic, in which low bone-mass and micro-architectural deterioration of bone tissue leads to increasing bone fragility and fracture. Vertebral and hip fractures lead to increased mortality, resulting in enormous health care costs. BMD testing by DEXA is used in diagnosis of osteoporosis. However, low-and middle-income populations are unable to conduct periodic examinations of bone mineral status. Thus, current study is mainly aimed at finding a cost-effective diagnostic-marker for osteoporosis. 170 participants, of whom 51 had osteoporosis, 62 had osteopenia and 57 had normal bone-mass. Selection of individuals was based on DEXA scan BMD. Sclerostin was determined by ELISA. The variables were compared using ANOVA test and ROC analysis was performed. Sclerostin levels were significantly decreased in osteoporosis (4.62 ± 1.6 ng/mL) and osteopenia (4.92 ± 1.4 ng/mL) compared with controls (5.74 ± 1.3 ng/mL), (p < 0.0001). Sclerostin level 5.6 ng/mL is the cut-off value for diagnostic purpose, according to good sensitivity and specificity. In patients with osteopenia and osteoporosis, decreased sclerostin levels were associated with an increased disease risk. These relationships were independent of BMD and bone turnover, suggesting that Sclerostin levels may reflect disease-severity in osteoporosis. Sclerostin measurements could become a useful clinical index for diagnosis of osteoporosis.

## Background:

Osteoporosis is asymptomatic until it becomes complicated by fractures [1]. Recently it has become a major clinical problem due to lifestyle modifications and dietary habits; the individuals with osteoporosis are at an increased risk of fractures resulting in major health and economic impact of the disease [2]. Dual energy X-ray absorptiometry (DXA) scan for identification of bone mineral density is the gold standard to diagnose osteoporosis. The DXA scan is a costly method to assess BMD for diagnosis of osteoporosis in poor, low- and middle-income peoples [3]. And it is only detected with BMD in the specific region of bone tissue not in the whole-body bones. The study aims to concentrate on a simpler and more cost-effective biochemical method for identification of osteoporosis. Therefore, our research community is increasingly interested in the analysis of biochemical markers for cost-effective measurement of bone mineral density. BMD levels were regulated by osteoclasts, osteoblasts and osteocytes. Osteocyte secretes sclerostin, which is an inhibitor of the Wnt/β-catenin pathway and thus inhibits the function, differentiation and survival of osteoblasts. Osteoblasts are responsible for bone mineralization but the above said inhibitory pathway may affect the normal bone remodelling process in osteoporosis [4]. Therefore, serum sclerostin might be a choice for diagnosis of osteoporosis. Therefore, it is of interest to assess the diagnostic role of serum sclerostin in patients with osteoporosis.

## Methodology:

This case control study was carried out at a tertiary care teaching hospital in India. The study approval was obtained from the Institutional Research Ethics Committee and a written informed consent was obtained from all the study participants. The participants answered a self-assessed questionnaire which contains details of age, gender, marital status, detailed history of demography, diet, exercise, smoking, alcohol consumption, menstrual history, medication and history of previous fractures and family history of bone disease were taken.

## Inclusion criteria:

170 participants in the age group between 30-90 years of both sexes, who have undergone DEXA scan for suspected osteoporosis and patients who come with bone fractures to the Orthopaedic Department of Sri Ramachandra Medical Centre, were chosen for the study.

## Exclusion criteria:

Patients with chronic disease, rheumatoid arthritis, ankylosing spondylitis, hyperparathyroidism, chronic smokers and alcoholics, patients on drugs like steroids, Immunosuppressive therapy, antiepileptics, bisphosphonates, hormone replacement therapy, vitamin-D, calcitonin and teriparatide were excluded from the study.

The height and weight was recorded in all the study participants and the body mass index (BMI) were calculated by dividing weight (kg) by height (m2). The waist and hip circumference was measured by standard method using stretch resistant measuring tape with 100g tension and waist-to-hip ratio was calculated by waist measurement (inches) divided by hip measurement (inches).

Bone mineral density (BMD) was determined at neck of femur (hip) and lumbar spine (L1-L4) by dual energy X-ray absorptiometry (DEXA) densitometer (GE Lunar Prodigy, Advance Bone Densitometer, USA). The DEXA scan was obtained by standard procedure according to manufacturer supplied protocol for scanning and analysis. All the BMD measurements were carried out by the same well-trained technician and same instrument for all the study participants. Daily quality control (QC) check was carried out by the measurement of Lunar Phantom. In this every day QC check provides stable results. The BMD values are expressed as the amount of bone mineral matter per cm2 area and obtained values.

A world Health Organization criterion for osteoporosis is defined based on the following bone density levels:

[1] T-score within 1 SD (+1 or -1) - Normal bone density.

[2] T-score of 1 to 2.5 SD (-1 to -2.5 SD) indicates low bone mass (Osteopenia).

[3] T-score of 2.5 SD or more (more than -2.5 SD) indicates the presence of osteoporosis

After obtaining the DEXA scan reports. The study subjects are grouped into three, based on the DEXA T score of BMD, according to WHO criteria,

[1] Group I - Normal bone mass (n=57)

[2] Group II - Osteopenia (n=62)

[3] Group III - Osteoporosis (n=51)

## Estimation of serum sclerostin:

2.5 ml of venous blood was collected without any anticoagulant (plain tube) for separation of serum. After separation the obtained serum sample was used to estimate the sclerostin level by commercially available enzyme linked immunosorbent assay kit (Elabscience, ELISA kit) according to the manufacturer protocol. The measurement range was 0.062 - 4 ng/mL and the analytical sensitivity was 0.062 ng/mL.

Statistical analysis was performed using SPSS version 20 (IBM SPSS V20). The continuous variables are expressed as mean ± SD. Comparison of normally distributed independent variables was performed using one way ANOVA with Tukey's HSD post hoc test for identification of differences between specific groups. Receiver operating characteristics (ROC) curve analysis was performed for determination of cut-off point by sensitivity and specificity. The levels of p<0.05 was considered statistically significant.

## Results and Discussion:

The strength of the bone can be described with mineral density of bone is 70% and quality of bone is 20%. This is a generally used method for easy to measure BMD, but, in clinical settings, quality of bone is not measurable yet [1]. The diagnosis of osteoporosis is established by the measurement of BMD or by the occurrence of a fragility fracture of the hip or vertebra or in the absence of major trauma [5]. In our study participants BMD of three groups (control, osteopenia and osteoporosis) were represented in Table 1. The neck of femur BMD (p<0.0001), lumbar spine BMD (p<0.0001) and T-score (p<0.0001) were significant variations observed between the three groups. Osteopenia and osteoporosis groups were lower BMD than the control group. Among the 170 participants, the percentage distribution of normal bone mass, osteopenia and osteoporosis groups were 33.5%, 36.5% and 30% respectively. Based on this observation we found that 66.5% of participants were diseased with low bone mineral density and 34.5% participants had normal BMD (Table 1).

Serum sclerostin is an osteocyte activity marker which regulates the formation of bone by declining the signaling of the Wnt pathway. Thereby decreased circulating sclerostin levels in the patients with high bone mass and increasing high sclerostin levels were strongly associated with high propensity of fracture risk [6]. After adjustment for age and other confounders, the relative fracture risk was more than sevenfold among postmenopausal women for each 1-SD increment increase in sclerostin level. Women in the highest range of serum sclerostin levels had about a 15-fold highest risk in fracture [7]. The bone resorption markers such as "(plasma cross-linked C-terminal telopeptide of type I collagen ([β-CTX], urinary CTX [µ-CTX], and urinary N-telopeptide of type I collagen [µ-NTX])" high level circulation were predictive of osteoporosis-related fractures but at much lower hazard ratio (HR) of value (1.0) than that of serum sclerostin [8,9]. The relationship between serum circulating sclerostin levels and increased fracture risk was independently decided by the bone mineral density levels and other confounding risk factors. High sclerostin observations are a strong and independent risk factor for osteoporosis-related fractures among postmenopausal women [10].

Bart *et al*. mentioned that the osteoporosis-related fracture risk in a total of 707 participants with postmenopausal women from Saudi Arabia with a mean follow-up of 5.2 ± 1.3 years. In multivariate Cox proportional hazard regression models adjusted for confounding risk factors, for fracture risk contribution in postmenopausal women was increased 47-fold for each 1-SD increase in sclerostin level, with women in the highest sclerostin level quartile having a nearly 15-fold high in fracture risk [11]. Similar to the results seen in the study of Arasu *et al*, serum sclerostin levels also associated with BMD, and subjects with the highest sclerostin levels and lowest BMD were at greatest fracture risk [7].

In contrast with the above study, group III individuals in this study had low serum sclerostin of 4.62 ± 1.6 ng/mL compared with group I (5.74 ± 1.3 ng/mL) and group II (4.92 ± 1.4 ng/mL). The statistically significant level was p<0.0001 as shown in (Table 2). This decreasing circulated serum sclerostin levels in group III could be due to decreased sclerostin expression which might be the consequence of apoptosis of osteocytes and deficiency of estrogen or testosterone. Serum sclerostin levels affect BMD of both cortical and cancellous bone in the general population.

Based on the mechanical loading the osteocytes regulate bone mass and bone density. The lacunae and the canalicular network among osteocytes may act as mechanical strain amplifiers, in order to raise osteocyte sensitivity to mechanical loading. The exact site of signal detection is not known, and may reside within the osteocyte cell body or dendrites, or both. Sclerostin is an osteocyte marker which regulates skeletal mechanical loading. Areas of concentrated strain in the skeleton show decreased levels of circulating sclerostin. In various mechanical loading experiments showing decreased microgravity, both up-regulation of sclerostin and low levels of bone mineral density occur [12]. Clinically, the serum sclerostin level is increased in healthy adult males during bed rest. Sex steroids are important for bone growth and maintenance. Estrogen signaling affects osteocyte regulation of bone density. Deletion of the estrogen receptor-alpha results in decreased sensitivity to mechanical loading, and estrogen withdrawal induces osteocyte apoptosis. In men with idiopathic osteoporosis, circulating sclerostin levels correlate with estrogen exposure, possibly reflecting lower osteocyte cell mass or number. In elderly men treated with gonadotropin-releasing hormone (GnRH) and 17beta estradiol, serum sclerostin levels were reduced, while men treated with GnRH agonist and testosterone showed increased circulating sclerostin. Studies on circulating sclerostin levels and sex hormones have not yet clearly demonstrated that altered sclerostin levels are due to osteocyte expression [13]. Sclerostin is predominantly synthesized by osteocytes; a possible explanation of the lower levels of sclerostin in osteoporotic patients is the age-dependent reduction in osteocyte number/density and change in morphology. This alteration might result in a decreased functioning of the osteocyte, including the production of sclerostin [14].

In our study the three different cut-off levels were shown in Table 4 according to the levels of sensitivity and specificity. The first cut-off 5.6 ng/mL of serum sclerostin represents good sensitivity and specificity. The second cut-off 6.6 ng/mL serum sclerostin represents the high sensitivity and the third cut-off 4.8 ng/mL serum sclerostin represents the high specificity. Therefore, in our study we found that 5.6 ng/mL of serum sclerostin level is an ideal cut-off based on good sensitivity, specificity, positive and negative predictive value.

This suggests that sclerostin secretion, metabolism, and/or clearance are rather stable in the population studied [15] although we observed consistent minor changes in serum circulating sclerostin levels. Such minor fluctuations may reflect changes related to aging, but assay variability may contribute too. In this study, we standardized serum sclerostin measurements at each time point, suggesting that the changes in serum sclerostin are not only explained by its assay variability but may also be influenced by other biological variability including dietary, physical activity and/or other lifestyle factors. Further work is needed to explore these observations 16,17, 18,19,20.

## Conclusion:

Patients with osteopenia and osteoporosis have low circulating serum sclerostin levels and significantly decreased BMD. Serum sclerostin levels were primarily associated with BMD in various bone diseases. Pathogenesis of bone loss depending on the circulating serum sclerostin levels and osteoporosis risk is of great clinical relevance. Accordingly, it is possible that serum sclerostin measurements could become a useful clinical index, together with BMD, that will help to determine which patients need to be treated to prevent future fractures. Recent clinical research studies showed that lowering circulating serum sclerostin resulted in improved bone health in postmenopausal women, our study population with decreased serum circulating sclerostin resulting in poor bone health and osteocyte abnormality. Further studies are needed to examine whether such therapeutic approaches will reduce fracture risk.

## Financial support:

This research study was partly supported by Founder-Chancellor Shri. N.P.V Ramasamy Udayar Research Fellowship grants.

## Figures and Tables

**Figure 1:**
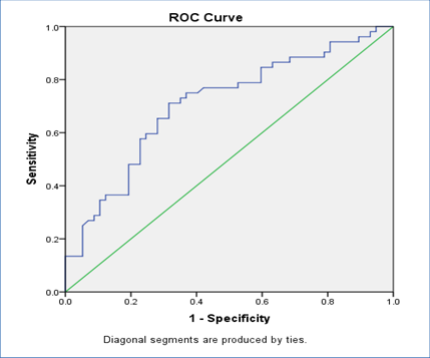
ROC curve of serum sclerostin level

**Table 1:** Age, gender and clinical presentation of study population

**Study population(n = 170)**		**Group - I Normal Bone Mass n = 57 (33.5%)**	**Group - II Osteopenia n = 62 (36.5%)**	**Group - III Osteoporosis n = 51 (30%)**
Age in years (Mean±SD)		52± 13	52± 12	55± 11
	Male	33 (19.4%)	34 (20%)	27 (15.9%)
Sex	Female	24 (14.1%)	28 (16.5%)	24 (14.1%)

**Table 2:** represents the physical, clinical and biochemical characteristics among three groups

**Characteristics**	**Group I(n=57) Normal bone mass (Mean ± SD)**	**Group II(n=62) Osteopenia (Mean ± SD)**	**Group III(n=51) Osteoporosis (Mean ± SD)**	**p value**	**Post hoc test **
NF BMD (g/cm2)	1.05 ± 0.95	0.872 ± 0.04	0.725 ± 0.06	< 0.0001**	B/W three groups
NF T score	0.01 ± 0.7	-1.3 ± 0.4	-2.7 ± 0.43	< 0.0001**	B/W three groups
LS BMD (g/cm2)	1.29 ± 0.11	0.971 ± 0.99	0.760 ± 0.92	< 0.0001**	B/W three groups
LS T score	0.4 ± 0.9	-1.3 ± 0.41	-2.8 ± 0.56	<0.0001**	B/W three groups
Height(cm)	162.8 ± 8.6	159.9 ± 8.3	157.9 ± 9.1	0.013*	I vs III
Weight(kg)	66.4 ± 8.7	67.4 ± 11.6	63.7 ± 10.6	0.164	NS
BMI (kg/m2)	24.9 ± 2	26.3 ± 4.2	25.5 ± 3.7	0.084	NS
Hip circumference(cm)	95.5 ± 9.4	104.1 ± 16.5	98.5 ± 12.5	0.002*	I vs II, I vs III
Waist circumference(cm)	87.8 ± 7.8	100.5 ± 14	94.1 ± 13.2	< 0.0001**	B/W three groups
W/H ratio	0.92 ± 0.02	0.98 ± 0.2	0.95 ± 0.03	0.033*	I vs II
Sclerostin (ng/mL)	5.74 ± 1.3	4.92 ± 1.4	4.62 ± 1.6	< 0.0001**	B/W three groups
*Represents statistically significant;
**Represents statistically highly significant

**Table 3:** cut-off levels of serum sclerostin

**Biomarkers**	**Cut-off**	**Sensitivity (%)**	**Specificity (%)**
Serum Sclerostin (ng/mL)	5.6	75	70
	6.6	85	40
	4.8	43	80

**Table 4:** The cut-off levels of different age groups and male & females

**Biomarkers**	**Age group(yrs)**	**Cut-off**	**Sensitivity (%)**	**Specificity (%)**
Serum Sclerostin (ng/mL)	30 - 45	5.6	75	70
	46 - 60	5.6	67	70
	> 60	5.6	76	80
	Male	5.6	80	70
	Female	5.6	60	80
